# Influence of genetic copy number variants of the human GLUT3 glucose transporter gene *SLC2A3* on protein expression, glycolysis and rheumatoid arthritis risk: A genetic replication study

**DOI:** 10.1016/j.ymgmr.2019.100470

**Published:** 2019-04-06

**Authors:** Kim R. Simpfendorfer, Wentian Li, Andrew Shih, Hongxiu Wen, Harini P. Kothari, Edward A. Einsidler, Arthur Wuster, Julie Hunkapiller, Timothy W. Behrens, Robert R. Graham, Michael J. Townsend, Doron M. Behar, Rui Hu, Elliott Greenspan, Peter K. Gregersen

**Affiliations:** a Robert S. Boas Center for Genomics and Human Genetics, the Feinstein Institute for Medical Research, 350 Community Drive, Manhasset, New York, USA; b Department of Molecular Medicine, Donald and Barbara Zucker School of Medicine at Hofstra/Northwell, New York, USA; c Department of Human Genetics, Genentech Inc., 1 DNA Way, South San Francisco, California, USA; d Department of Biomarker Discovery OMNI, Genentech Inc., 1 DNA Way, South San Francisco, California, USA; e Gene by Gene, Genomic Research Center, Houston, TX, USA

**Keywords:** GLUT3, *SLC2A3*, Glycolysis, Deletion, Rheumatoid arthritis, Copy number variant, Glucose transport

## Abstract

**Objectives:**

The gene encoding glucose transporter 3 (GLUT3, *SLC2A3*) is present in the human population at variable copy number. An overt disease phenotype of *SLC2A3* copy number variants has not been reported; however, deletion of *SLC2A3* has been previously reported to protect carriers from rheumatoid arthritis, implicating GLUT3 as a therapeutic target in rheumatoid arthritis. Here we aim to perform functional analysis of GLUT3 copy number variants in immune cells, and test the reported protective association of the GLUT3 copy number variants for rheumatoid arthritis in a genetic replication study.

**Methods:**

Cells from genotyped healthy controls were analyzed for *SLC2A3*/GLUT3 expression and glycolysis capacity. We genotyped the *SLC2A3* copy number variant in four independent cohorts of rheumatoid arthritis and controls and one cohort of multiple sclerosis and controls.

**Results:**

Heterozygous deletion of *SLC2A3* correlates directly with expression levels of GLUT3 and influences glycolysis rates in the human immune system. The frequency of the *SLC2A3* copy number variant is not different between rheumatoid arthritis, multiple sclerosis and control groups.

**Conclusions:**

Despite a robust *SLC2A3* gene copy number dependent phenotype, our study of large groups of rheumatoid arthritis cases and controls provides no evidence for rheumatoid arthritis disease protection in deletion carriers. These data emphasize the importance of well powered replication studies to confirm or refute genetic associations, particularly for relatively rare variants.

## Highlights

• T cell and macrophage expression of *SLC2A3*/GLUT3 correlates to *SLC2A3* gene copy number in a dose dependent manner.
• Glycolysis rates are reduced in individuals harboring a deletion of the GLUT3 gene *SLC2A3*
• Deletion of *SLC2A3* is not associated with protection from rheumatoid arthritis
• Deletion of *SLC2A3* is not associated with risk for multiple sclerosis
• GLUT3 is not a viable therapeutic target for RA as previously proposed based on a protective association of *SLC2A3* deletion.

## 1 Introduction

Distinct metabolic profiles characterize the main players in rheumatoid arthritis (RA) pathogenesis. For example, CD4+ effector T cells preferentially utilize glycolysis upon activation and upregulate their expression of glucose transporters GLUT1 and GLUT3, while induced regulatory T cells favor oxidative lipid metabolism [1,2]. The gene encoding GLUT3, *SLC2A3*, is copy number variable in humans [3] and heterozygous deletion is reported to protect carriers from developing RA [3]. A deletion or duplication of 129 kb at chromosome 12p13.31 results in variants of one or three (or more) total *SLC2A3* gene copies respectively. Heterozygous deletion at *SLC2A3* is consistently found in 0.5–1% of individuals, while one or more duplications are found in approximately 4% of normal subjects across multiple populations [3,4]. The protective association of *SLC2A3* for RA implicates GLUT3 as a potential therapeutic target in treatment or prevention of RA.

The emerging data on the metabolic changes that accompany immune activation prompted us to explore the effects of the human *SLC2A3* CNVs on the metabolism of immune cells that have been implicated in RA, as well as to replicate the previous association data.

## 2 Results

### 2.1 *SLC2A3* CNV genotype calling approach

**Fig. 1:**
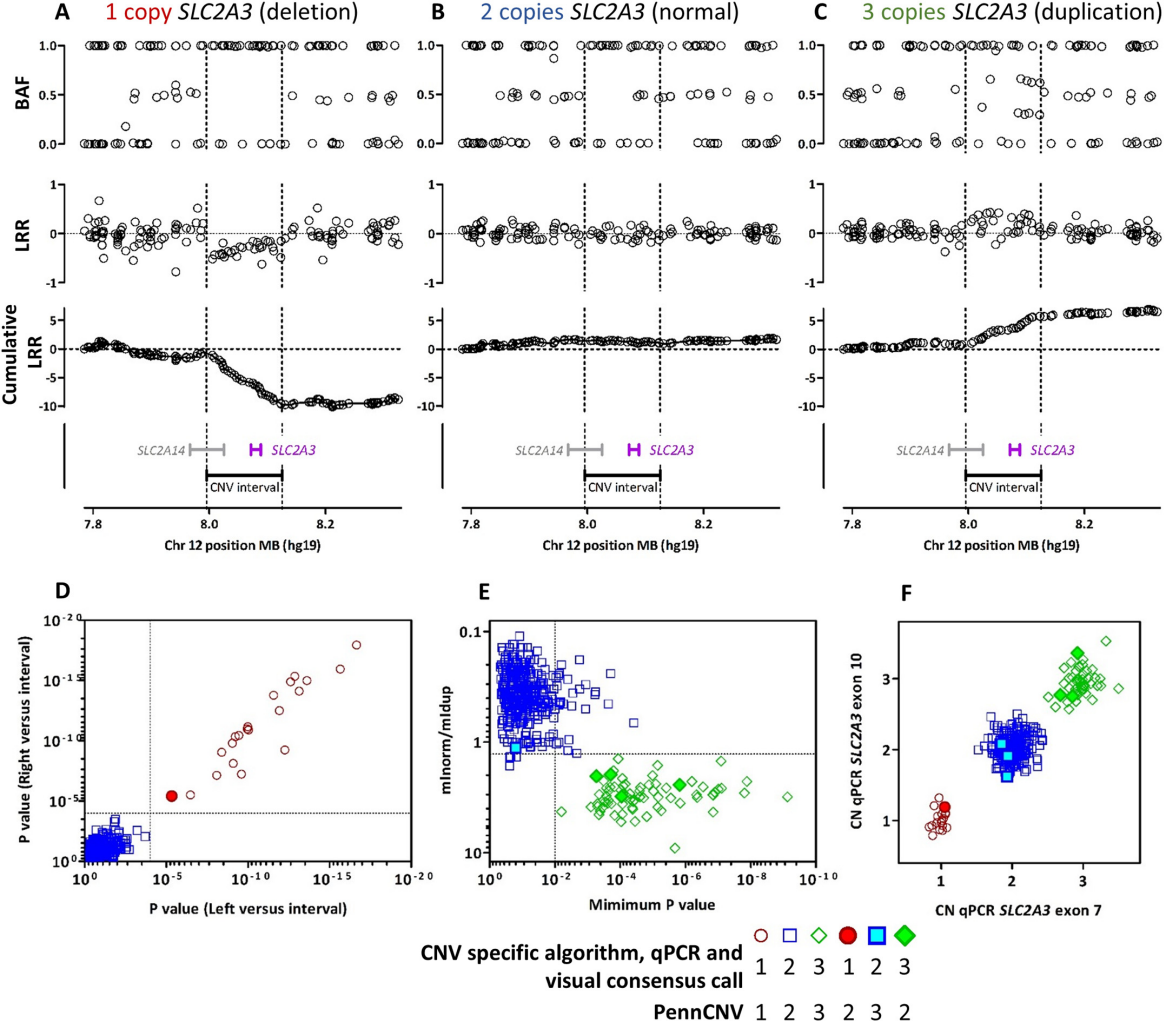
*SLC2A3* CNV genotyping. Representative images of data plots used for visual calls of copy number in a deletion carrier (A), normal subject (B) and a duplication carrier (C). Plots show BAF (top), LRR (middle) or cumulative [22] LRR (bottom) by chromosomal position. Vertical dotted lines depict the breakpoints of the known CNV interval and the positions of the *SLC2A3* and *SLC2A14* genes are depicted as indicated. The new CNV specific algorithm uses *P* value cutoffs and an expected BAF value fit ratio threshold to call *SLC2A3* copy number. *P* values of *t*-tests comparing LRR of SNPs inside the CNV breakpoints to LRR of SNPs 350 kb either side of the CNV interval (D) or minimum P value versus BAF value fit ratio (E). QPCR values of genomic DNA samples classified as 1, 2 or 3 copies by SNP array methods and visual calls.

Copy number variants (CNVs) can be challenging to accurately define, and accurate genotyping is best carried out by a variety of complementary methods. Here we focus on qPCR and estimates from SNP array data. To validate our genotyping methods we utilized GAP and NYCP control cohorts, described previously, for which we obtained SNP chip array values and DNA for typing via PCR methods [5,6]. Quantitative PCR (qPCR) revealed a number of false positive and false negative CNV calls by PennCNV [21] (tableS1, tableS2). By plotting B allele frequency (BAF) and logR ratios (LRR) we verified that visual inspection could corroborate qPCR CNV calls (Fig. 1A–C). In the GAP and NYCP control sets, all subjects that received discrepant PennCNV and qPCR CNV calls (or high LRR SD or NumCNV values) were inspected visually, and all visual calls agreed with qPCR genotypes, verifying that the mismatches were due to false negative or false positive PennCNV calls. We introduced a third method to estimate *SLC2A3* copy number from known CNV interval positions [3]. This method compared LRR values of SNPs inside the known CNV breakpoints, to values 350 kbps either side of the CNV interval (Fig. 1D) and calculated fit to expected BAF values within the CNV interval for a duplication, deletion or a normal diploid interval (Fig. 1E). Results from this method agreed with qPCR and visual inspection calls for all subjects in the GAP and NYCP datasets (Fig. 1F). Due to the nature of performing large genetic studies, it is not always possible to obtain both genomic DNA to perform qPCR, and SNP chip array values in order to confirm CNV calls by complementary methods. Our analysis here show that, in the absence of using qPCR to validate PennCNV calls, that combining PennCNV with visual inspection and a CNV specific calling method is sufficient for false calls by PennCNV to be detected and corrected for.

### 2.2 *SLC2A3* CNV frequencies in RA cases, MS cases and controls

**Fig. 2:**
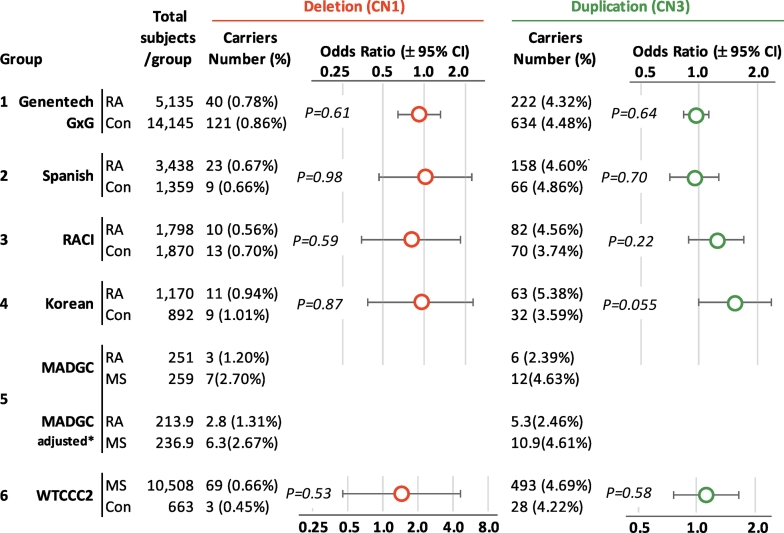
Genetic association of *SLC2A3* CNVs in RA cases, MS cases and controls. The number, and frequency, of deletion and duplication CNVs, and total subjects genotyped is given for each group of cases and controls. The odds ratios ±95% confidence intervals (error bars) are plotted on a log2 scale. Group details are supplied in online supplementary table S1. P values are from chi-square test statistic.

In an attempt to replicate the previous report of an association with RA susceptibility [3], to which our group contributed, we genotyped the *SLC2A3* CNV in four independent groups of RA cases and controls (tableS1)[7,8]. Comparisons of CNV frequencies between cases and controls within groups revealed no compelling evidence for either a risk or protective association, despite the large sample sizes (Fig. 2, tableS3). The sample sizes of these cohorts (1 to 4) ensured good power to replicate the initial protective associations reported in Swedish cases and controls [3] at 99.99%, 91.8%, 89.1% and 67.5% respectively (table s6). We tested for any effects of the CNVs on clinical covariates within RA cases and did not observe any effect of *SLC2A3* gene copy number on disease severity measures (figureS1).

We further tested the subgroups within each group (which differ by genotyping method or genotyping array) to confirm the validity of combining the groups for association testing (tableS7). Only the Korean control subgroups showed evidence for significant heterogeneity. We note that the relatively small size may explain this result for the Korean control groups. We also genotyped the *SLC2A3* CNV in 450 families with autoimmune disease from the Multiple Autoimmune Disease Genetic Consortium (MADGC) to look for association with RA or autoimmune disease in general [9]. As shown in Fig. 2, we observed that the deletion in RA patients was not less than the observed frequencies in controls. Interestingly, within the MADGC family members affected with multiple sclerosis (MS) we observed significant enrichment of the deletion at 2.7%. To follow on from this suggestive deleterious association, we analyzed the *SLC2A3* CNV in 10,667 MS cases from the Wellcome Trust Case Control Consortium (WTCCC) [10]. The deletion was observed at a frequency of 0.66%, a frequency not significantly different than matched controls, or observed in any of our other control datasets.

### 2.3 *SLC2A3* CNV genotype correlates with expression level and glycolysis

**Fig. 3:**
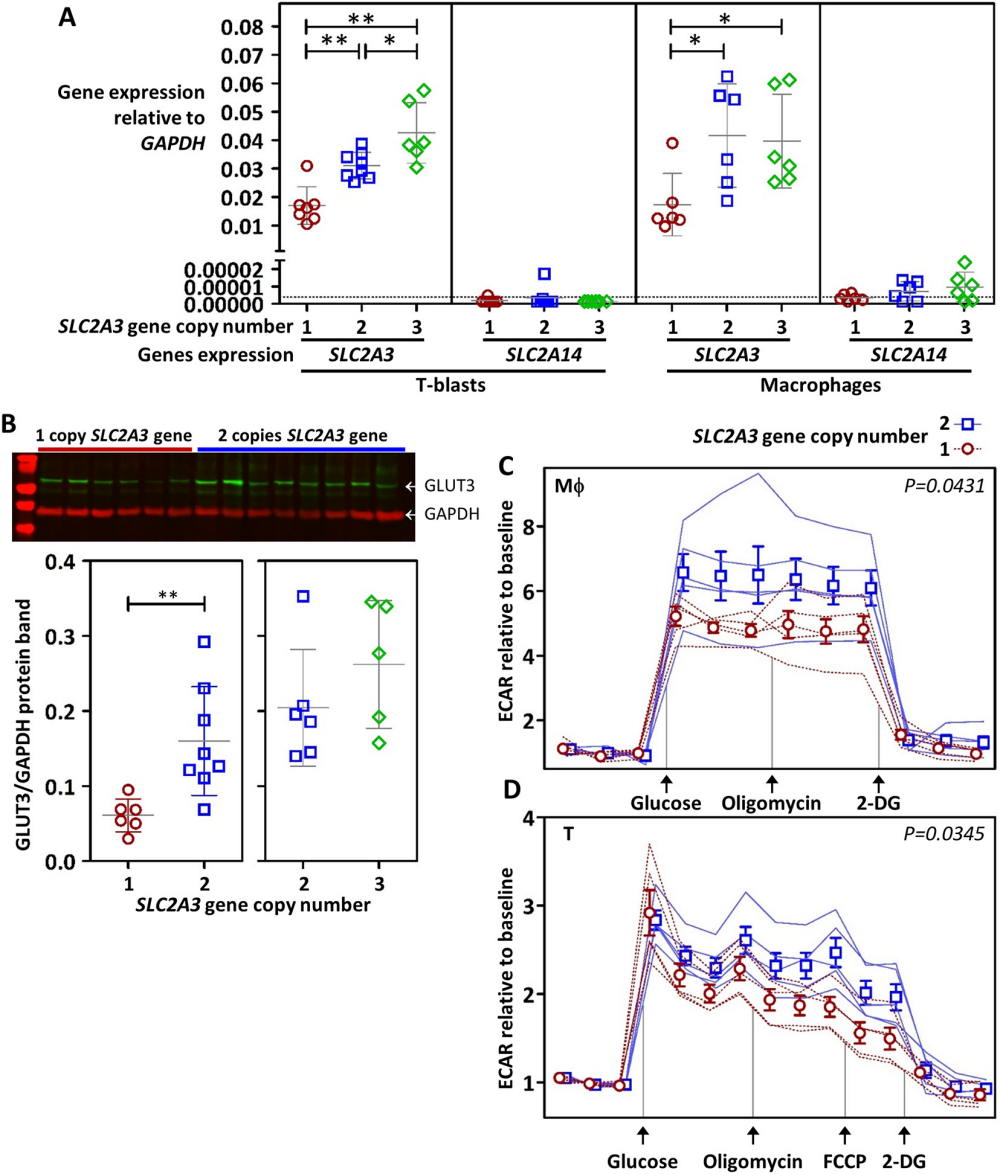
Correlation of GLUT3 expression and glycolysis to *SLC2A3* gene copy number. The level of *SLC2A3* and *SLC2A14* transcript (A) in genotyped control subject T cells activated with anti-CD3/anti-CD28 coated beads (T-blasts 10 days) and in monocytes differentiated into macrophages with M-CSF (14 days). B) GLUT3 protein levels in T-blasts (day 10) detected with anti-GLUT3 (EPR10508(N)) rabbit monoclonal antibody. Glycolysis measured as extracellular acidification rates (ECAR) in genotyped control subject's LPS activated macrophages (14 days) (extracellular acidification occurs predominately from the excretion of lactic acid after its conversion from pyruvate during glycolysis) (C) and PMA and ionomycin activated T-blasts (7 days) (D). The level of detection is indicated by the dotted line in A. In A and B each symbol represents one unique control subject, horizontal lines indicate the mean ± SD. In C and D each line represents one unique control subject and symbols represent the mean ± SEM for each genotype group, groups are nudged to prevent symbol overlap. Statistical significance of expression and protein data was tested using a two-tailed Mann-Whitney *U* test and is denoted as *p < .05, **p < .01, ***p < .001. Statistical significance of ECAR data (*P* values shown) was calculated by unpaired *t*-test of AUC (one-tailed). Genotyped subjects of *SLC2A3* copy number 1 are circles, copy number 2 are squares and copy number 3 are diamonds. As previously reported we noted that GLUT3 protein aggregates to form high molecular weight smears in Western blot of boiled lysate and that copy number dependent protein levels at 54 kDa could only be observed in the absence of boiling (see online supplementary fig. S5).

As these genetic data were being developed, we endeavored to demonstrate whether GLUT3 deletions have an effect on immune metabolism. GLUT3 expression is reported in monocytes and T lymphocytes in the human immune system [11], and in activated T lymphocytes in the murine immune system [1]. To confirm strong expression of GLUT3 protein in the human immune system we isolated and activated major cell subsets. Despite evidence for expression of *SLC2A3* transcript in human neutrophils [12] we were unable to detect robust expression of GLUT3 protein in resting or activated neutrophils. Consistent with the non-glycolytic state of quiescent cells, GLUT3 protein was absent or very weak in the absence of activating factors [13], and strongly induced following activation. The strongest expression of GLUT3 protein was observed in monocyte derived macrophages and blasting T lymphocytes (figureS2). We isolated T cells and monocytes from PBMC of genotyped controls and cultured to T-blasts and macrophages to compare transcript and protein expression by *SLC2A3* gene copy number. We observed a significant reduction of GLUT3 expression at both the transcript and protein level in subjects carrying the deletion (Fig. 3A–B) compared with subjects with 2 or 3 copies of the gene. In T-blasts we observed an increase in *SLC2A3* transcript of subjects carrying 3 copies of the *SLC2A3* gene, compared with subjects with 2 copies, however, we were unable to detect a similar increase at the protein level. Notably, GLUT14 protein is unlikely to be present at functional levels as expression of *SLC2A14* transcript was undetectable in most samples, and at its highest level, *SLC2A14* is >2000 fold lower than expression of *SLC2A3*.

We also measured expression of the GLUT1 gene transcript (*SLC2A1*) and expression of genes neighboring the CNV locus. The expression of *CLEC4C* and *FOXJ2* is not affected by the *SLC2A3* CNVs, but reduced expression of *NANOG* was observed in T cells of deletion carriers. However, the effect is unlikely to disrupt T cell function as expression of *SLC2A3* is at least 50 fold greater than the expression of *NANOG* (figureS3).

To determine the effect of altered GLUT3 expression levels on metabolic functions we measured the extracellular acidification rate (ECAR) as a proxy of glycolysis of activated T-blasts and macrophages. We observed that both cell types reached maximal glycolysis rates in the absence of mitochondrial inhibition, and that ECAR was lower in deletion carriers than subjects with two copies of *SLC2A3* (Figs. 3C–D).

## 3 Discussion

Prompted by a previous study of protection from RA, we explored the biological effects of *SLC2A3* deletion on immune cell function. The results clearly indicate that the presence of a heterozygous *SLC2A3* deletion correlates with reduced glycolysis rates and a reduction of GLUT3 expression in both T cells and macrophages of individuals carrying this genotype. However, the protective association with RA (OR~0.5) was not confirmed (combined OR = 0.87[0.67–1.14]). We also did not observe evidence of an association with multiple sclerosis, despite initial encouraging results in multiplex families with autoimmunity.

There are a number of possible explanations of why this study did not replicate the previously reported association of protection from RA with the *SLC2A3* deletion. Foremost, we note that our current data reveal that the number of PennCNV false calls can be significant (tableS4). By their nature, random sampling of rare events can lead to false differences more often than common events [14]. We analyzed 15 groups of cases and controls of European ancestry and the highest frequency of the deletion that we observed was of 1.4% in 712 RA cases. In comparison, the frequency of the deletion reported in Swedish controls in the original study was 2.6%. In comparison, the frequency of the deletion reported in RA cases was 1.17%, which is not less than the frequency reported in their US or UK controls, or any of our control groups of European ancestry.

Our result is consistent with two previous genome-wide CNV association studies that also found no association of the *SLC2A3* CNV with RA [15,16], however, we note that these studies may have been underpowered to detect a statistically significant difference in frequency of the uncommon *SLC2A3* CNV.

Maintenance of *SLC2A3* CNVs in the human population at fairly constant frequencies between ethnically diverse groups (Fig. 2, tableS5) suggests selective advantages may exist for these variants. The reduced expression of GLUT3 associated with genetic deletion of *SLC2A3* could offer some level of protection to the host from infection with Chlamydia bacteria [17] and blood-stage parasites that rely on GLUT3 from their host cells for glucose metabolism [18].

In summary these results confirm that deletion of *SLC2A3* correlates directly with expression levels of GLUT3 and glycolysis rates in the human immune system, but are not associated with protection from RA or susceptibility to MS. Our improved method of CNV allele calling should permit future exploration of whether *SLC2A3* CNVs have an impact on other immune phenotypes such as susceptibility to infection. In addition, since associations of *SLC2A3* with several CNS phenotypes has been suggested [19,20], our methods should also be applied to the emerging large GWAS datasets of these disorders.

## Collaborators

WTCCC MS and control data was obtained from European Genome-phenome Archive [10]. Chip genotyping array data of Spanish RA cases and/or Spanish controls was shared with us by Martin Kerick and Javier Martin (Instituto de Parasitología y Biomedicina ‘López-Neyra’, CSIC, PTS Granada, Granada, Spain), Raquel López-Mejias and Miguel Angel González-Gay (Epidemiology, Genetics and Atherosclerosis Research Group on Systemic Inflammatory Diseases, IDIVAL and School of Medicine, University of Cantabria, Santander, Spain).

## Contributors

K.R.S. and P.K.G. designed the study, and wrote the manuscript. W.L. and K.R.S. designed the CNV specific calling methodology. W.L. ran PennCNV, wrote and ran the CNV specific algorithm script, and plotted BAF, LRR and LRR cumulative for visual calling. A.S. contributed to dataset management and statistical analysis. K.R.S. performed CNV visual calls, CNV data analysis, IBD and ancestry PCA. H.W. and H.P.K. performed functional studies. H.P.K. and K.R.S. performed qPCR genotyping. K.R.S. and E.A.E. analyzed the MADGC dataset and performed statistical correction for relatedness. All authors contributed to discussion.

## Funding

This work was supported by the Pfizer ASPIRE Research Award WI215050 (USA). K.R.S is supported by the NIH CDA K01AR071502 (USA). PKG is supported by NIH grant UH2AR067694 (USA).

## Appendix A Supplementary data

Supplementary material Image 1
