# Supplementary material for: Influence of genetic copy number variants of the human GLUT3 glucose transporter gene SLC2A3 on protein expression, glycolysis and rheumatoid arthritis risk: A genetic replication study
Source: Mol Genet Metab Rep. 2019 Apr 6;19:100470. doi: 10.1016/j.ymgmr.2019.100470 (PMC6453668; doi:10.1016/j.ymgmr.2019.100470)

Genetic copy number variants of the human GLUT3 glucose transporter gene *SLC2A3* influence protein expression and glycolysis in T cells and monocytes, but do not influence risk for rheumatoid arthritis.

Kim R. Simpfendorfer, Wentian Li, Andrew Shih, Hongxiu Wen, Harini P. Kothari, Edward A. Einsidler, Arthur Wuster, Julie Hunkapiller, Timothy W. Behrens, Robert R. Graham, Michael J. Townsend, Doron Behar, Rui Hu, Elliott Greenspan, Peter K. Gregersen

Supplementary Material

MATERIALS & METHODS

Subject and material details

GAP controls cryopreserved PBMC (~10million PBMC/vial) or fresh blood (for neutrophil isolation, drawn into BD Vacutainer EDTA tubes) were collected from de-identified subjects in the Tissue Donation Program at the Feinstein Institute for Medical Research through an IRB-approved protocol; 'Genotype and Phenotype (GAP) Registry (IRB# 09-081), a national resource for genotype-phenotype studies'. Informed consent was obtained from GAP subjects. Of genotyped GAP subjects whose PBMC were used in this study, 73% were female. All PBMC of GAP subjects copy number 1 or copy number 3 were gender matched to a copy number 2 subject within the experiment. Age was matched as best as possible (average 3.5 year age gap). Ethnicity was matched for 88% of matched pairs. The average age of subjects was 57 years (range 26-91 years of age). Non-genotyped PBMCs used in immune subset study of GLUT3 protein expression were from leukopacs of New York Blood Center de-identified donors. RA cases, MS cases and controls cohorts source and references are given in Table S1.

Table S1. Source of cases and controls for genetic replication study.

Group	Cohort	Group	Sub group	Chip	Taq man qPCR	Illumina Platform	Number subjects passed QC	Source / Ref
1	Genentech	RA cases	**Sum of Genentech RA cases**				**5,135**	Genentech Inc., South San Francisco, CA
			a	✓		Human Omni 1 Quad v1	1,965	
			b	✓		HumanOmni 2.5 8v1	306	
			c	✓		HumanOmni 2.5 8v1	1,942	
			d	✓		Human Hap 550 v3	712	
			e	✓		Human 1M Duo v3	210	
	GxG	Controls	**Sum of Gene By Gene controls**				**14,145**	Gene by Gene, LTD., Houston, TX. *Note: Files already filtered for QC and European ancestry prior to CNV analysis
			a	✓		GxG Comprehensive Array v1	11,075	
			b	✓		GxG Comprehensive Array v1	3,070	
2	Spanish	RA cases	**Sum of Spanish RA cases**				**3438**	Institute of Parasitology and Biomedicine López-Neyra, CSIC, Granada, Spain. Epidemiology, Genetics and Atherosclerosis Research Group on Systemic Inflammatory Diseases IDIVAL, Santander, Spain.
			a	✓		Human Hap 300 v1	2,702	
			b	✓		Human Hap 300 v1	736	
		Control	**Sum of Spanish controls**				**1359**	
			a	✓		Human Hap 300 v1	1,010	
			b	✓		Human 1M Duo v3	349	
3	RACI	RA cases	-		✓	-	**1,798**	As described Okada Y, Wu D, Trynka G, et al. Genetics of rheumatoid arthritis contributes to biology and drug discovery. *Nature.* Feb 20 2014;506(7488):376-381.
		Controls	-		✓	-	**1,870**	
4	Korean	RA cases	**Sum of Korean RA cases**				**1,170**	As described in Freudenberg J, Lee HS, Han BG, et al. Genome-wide association study of rheumatoid arthritis in Koreans: population-specific loci as well as overlap with European susceptibility loci. *Arthritis Rheum.* 2011;63(4):884-893.
			a	✓	✓	Human 610 Quad v1	793	
			b	✓		Human 610 Quad v1	12	
			c		✓	-	365	
		Controls	**Sum of Korean controls**				**892**	
			a	✓	✓	Human 610 Quad v1	380	
			b	✓		Human 610 Quad v1	112	
			c		✓	-	400	
5	MADGC	RA cases	-	✓	✓	**Global Screening Array 24 v1**	**259**	As described in: Criswell LA, Pfeiffer KA, Lum RF, et al. Analysis of families in the multiple autoimmune disease genetics consortium (MADGC) collection: the PTPN22 620W allele associates with multiple autoimmune phenotypes. *Am J Hum Genet*. 2005;76(4):561-571.
		MS Cases	-	✓	✓	**Global Screening Array 24 v1**	**260**	
6	WTCCC2	MS Cases	-	✓		**Human 670 Quad Custom v1**	**10,508**	As described in Reference 10: Sawcer S, Hellenthal G, Pirinen M, et al. Genetic risk and a primary role for cell-mediated immune mechanisms in multiple sclerosis. *Nature.* 2011;476(7359):214-219.
		Controls	-	✓		**Human 670 Quad Custom v1**	**663**	

Recombinant protein expression in HEK293T cells

Expression constructs were purified from E.coli (BL21-Gold, Agilent technologies, #230132) by Maxi-Prep (GeneJET Endo-Free Plasmid Maxiprep Kit, ThermoFisher Scientific, #K0861). HEK293T (source; female, ATCC, CRL-3216) were cultured in DMEM media supplemented with 10% FBS and Penicillin/Streptomycin at 37°C, 5% CO_2 for up to 12 passages. HEK293T cells were grown to 90% confluence in 6 well plates the day before the transfection. HEK-293T cells were transfected with 4ug plasmid DNA using Lipofectamine (Invitrogen) at 1:2.5 ratio. Transfected cells were sorted for positive GFP expression. TurboGFP tagged expression constructs from Origene: Glucose Transporter GLUT1 (SLC2A1, NM_006516 Human Tagged ORF Clone in pCMV6-AC-GFP, #RG222696), Glucose Transporter GLUT3 (SLC2A3, NM_006931 Human Tagged ORF Clone in pCMV6-AC-GFP, #RG204430), SLC2A14 (NM_153449, Human Tagged ORF Clone in pCMV6-AC-GFP, #RG216671), pCMV6-AC-GFP Tagged Cloning Vector (turbo GFP, #PS100010).

Primary human leukocyte culture, activation and differentiation

To culture T blasts and differentiate macrophages from CNV genotyped subjects, T cells and monocytes were enriched from total PBMC by adherence/non-adherence. Thawed PBMCs were seeded (5 million cells/dish) in 35x10mm dishes and incubated with RPMI medium supplemented with 10% FBS, 1X Penicillin/Streptomycin and 2mM glutamine at 37°C, 5% CO2 for 4 to 6 hours. Non-adherent cells in supernatant (containing T cells) were collected and cultured in RPMI1640 media supplemented with 10% FBS, 1X Penicillin/Streptomycin and 2mM glutamine and expanded to T-blasts with anti-CD3/anti-CD28 beads (Dynabeads ThermoFisher Scientific #11131D) and recombinant human IL-2 (10ng/ml Peprotech #200-02). Fresh IL-2 was added every 3 days of culture. Cells were cultured for times indicated in figure legends. Adherent cells (containing monocytes) received fresh RPMI medium supplemented with 10%FBS, 1X Penicillin/Streptomycin and 2mM glutamine and M-CSF (25ng/ml) every 3 days.

To generate T cells used in Figure S4, T cells were purified from frozen PBMC with EasySep Human T Cell Enrichment Kit (STEMCELL, #19051). Cells were harvested immediately, or following activation with anti-CD3/anti-CD28 beads and IL-2 (10ng/mL) for indicated times.

To isolate monocytes and differentiate macrophages, monocytes were purified from frozen PBMC using EasySep Human Monocyte Enrichment Kit (STEMCELL, Cat#19059). Monocytes were differentiated to macrophages by culturing with M-CSF (25ng/ml, Human Recombinant M-CSF, Stem cell technology, #78057.1) for 7 days. Macrophages were activated with LPS (10ng/ml).

B cells were purified from frozen PBMC with EasySep Human B cell Isolation Kit (STEMCELL, #17954) and are activated with anti-IgM (2000ng/ml, Southern Biotech, #2020-01), anti-CD40 (1000ng/ml, MAB89 Beckman Coulter, #IM1374), IL-4 (50ng/ml, PeproTech, #200-04) and IL-21(100ng/ml, PeproTech, #200-21) for indicated times.

NK cells were isolated from frozen PBMC with EasySep Human NK cell Enrichment Kit (STEMCELL, #19055); IL-2 (10ng/ml Peprotech #200-02) was added for activation.

Neutrophils were isolated from whole blood by immunomagnetic negative selection with EasySep Direct Human Neutrophil Isolation Kit (STEMCELL, #19666) and activated by GM-CSF (65ng/ml, Human Recombinant GM-CSF, STEMCELL, #78015.1), LPS (10ng/ml, Sigma-Aldrich, #L4524) or fMLP (100nM, Sigma-Aldrich, #47729).

Genotyping qPCR

CNV genotyping of 2-10ng genomic DNA were performed in duplex 384 well qPCR assays combining target assay with RNAseP reference assay using the Viia7 Real-Time PCR System (A&B Applied Biosystem). Copy number was calculated from normalized delta CT as 2X the fold change of target assay CT to reference assay CT values. CNV genotyping assays from ThermoFisher Scientific: Taqman custom paralogue ratio SLC2A3 CNV assay (SLC2A3 probe VIC: SLC2A14 probe FAM, Cat#AH1SEM4), Taqman CNV Assay 3' of SLC2A3 exon 10 (Chr.12:7919192 on GRCh38, Cat#4400291, Assay ID: Hs03830809), Taqman CNV Assay 5' of SLC2A3 intron 6 (Chr.12:7925991 on GRCh38, Cat#4400291, Assay ID: Hs04408256), Taqman CNV Assay 5' of SLC2A3 upstream of exon 1 (Chr.12:7936561 on GRCh38, Cat#4400291, Assay ID: Hs03826116), TaqMan™ Copy Number Reference Assay (human RNase P, Cat#4403328).

Gene expression qPCR

RNA extraction was performed using Trizol reagent and corresponding extraction protocol. Glycogen carrier protein was added to samples with less than 1 million cells. Reverse transcription was performed with High-Capacity cDNA Reverse Transcription Kit as per the manufacturers protocol with up to 1,800ng RNA template. One microliter cDNA per well was used as template in 384 well singleplex qPCR assays using the Viia7 Real-Time PCR System (A&B Applied Biosystem). Gene expression fold change expression values were calculated from CT values of target and indicated housekeeping genes. ThermoFisher Scientific Taqman gene expression assays: SLC2A3 (#4331182, Assay ID: Hs00359840_m1), SLC2A1 (#4331182, Assay ID: Hs00892681_m1), SLC2A14 (#4331182, Assay ID: Hs03044883_m1), NANOG (#4331182, Assay ID: Hs02387400_g1), GAPDH Endogenous Control (#4333764F), CLEC4C (#4351372, Assay ID: Hs01092460_m1), FOXJ2 (Cat#4331182, Assay ID: Hs00218236_m1), Human HPRT1 (HGPRT Endogenous Control, #4448484, Assay ID: Hs99999909_m1).

Protein lysate and WB

Transfected GFP+ HEK293T cells were lysed with RIPA buffer (ThermoFisher Scientific, #89900) at a concentration of $1x10^6$ cells/150uL. Human primary and cultured leukocytes were lysed with RIPA buffer at $a1x10^6$/25~50ul. Lysates were prepared with denaturing loading buffer but were not boiled prior to loading unless specified in the figure legend. Western blot was performed with either 10% or 4-20% protein gel and corresponding systems as indicated in the figure legends (NuPAGE™ 10% Bis-Tris Protein Gels, #NP0316 or 4–20% Mini-PROTEAN® TGX™ Precast Protein Gels, Biorad, #4561096). Following blocking, antibodies were applied to membranes in a solution of 20% blocking buffer, 80% PBS. Primary antibodies: rabbit monoclonal anti-Glucose Transporter GLUT3 [EPR10508(N)] (Abcam Ab191071), mouse anti GAPDH (Sigma-Aldrich #SAB1403850), mouse anti-turboGFP (clone OTI2H8 Origene #TA150041), rabbit anti-GLUT1 (Abcam #Ab32551), rabbit polyclonal anti-GLUT3 (Proteintech #20403-1-AP). Secondary antibodies: donkey anti-Rabbit IgG (H + L) (IRDye® 800CW LI-COR Biosciences #925-32213), goat anti-Mouse IgG (H + L) (IRDye® 680RD LI-COR Biosciences #925-68070). Western blot band quantification was performed using AzureSpot rolling ball background correction (AzureSpot 2.0, Azure

Biosystems). Band quantification is plotted as ratio of target band to GAPDH band (both volume minus background values).

Glycolytic rate assay

Macrophages or T-blasts were adhered to assay plate wells using Cell-Tak (Corning, #354240). Macrophages were rested overnight and activated with LPS (10ng/ml) for 2 hours prior to assay. T-blasts were activated with PMA (500ng/ml, Sigma-Aldrich, #P8139) and Ionomycin (500ng/ml, Sigma-Aldrich, #I9657) for 4 hours prior to seeding into the Seahorse plate, then rested for one hour prior to the Seahorse assay (Seahorse XFp Analyzer, Agilent Technologies).

Quantification and Statistical Analysis

PennCNV: A hidden Markov model based CNV detection program, PennCNV (version 1.0.3), was downloaded from penncnv.openbioinformatics.org. We modified the Perl scripts from PennCNV to be able to read gzip compressed files and to recognize other missing data symbols. An older version of Perl was used to accommodate the compatibility issue. The hhall.hmm hidden Markov model parameter file was used.

The *SLC2A3* CNV specific genotype calling method: calculated several values from chromosome 12 LRR and BAF. The CNV interval was delimited as known breakpoints (hg19 Chr12:7,995,630 – 8,125,390) ± 350kb flanking regions.

Using LRR values; (1) LRR values of flanking SNPs were compared to SNPs within the CNV breakpoints by a t-test (arbitrary precision R code: exp(pt(abs(t), df, lower.tail=F, log.p=T), where df is the degrees of freedom for Student's t test, to generate 'pv.left' and 'pv.right', (2) Average LRR values from flanking regions were determined to be greater than or less than average LRR values within the CNV boundaries to generate 'sign.left' and 'sign.right' values.

Using BAF values within the CNV boundaries; (1) The number of SNPs with BAF=0.5±0.05 is given as 'nHalf2'; (2) a set of BAF values was defined for deletion with genotype AA and BB as: BAF(del)=(0,1), mIdel is the sum of absolute distances between BAF and the nearest BAF(del). Or, $= \sum_{i=1}^{n} |BAF_i - cloest\ BAF(del)|$, where index i goes through all samples (1,2,….n); (3) a set of BAF values was defined for normal two copy genotypes AA,AB,BB as BAF(norm(=(0, 0.5, 1), mInorm is the sum of absolute distance between BAF and its closest BAF(norm); (4) a set of BAF values was defined for a duplication genotype with AAA, AAB, ABB, BBB as BAF(dup)=(0,1/3, 2/3, 1), mIdup is the sum of absolute distance between BAF and its closest BAF(dup) value.

For GAP, to call the copy number genotype as a deletion (copy number = 1) these criteria must be met: sign.left=-1, sign.right=-1, pv.left<0.01, pv.right<0.01, nHalf2 ≤1. For GAP, to call the copy number genotypes as a duplication (copy number =3) these criteria must be met: sign.left=+1, sign.right=+1, pv.left<0.05, pv.right<0.05, mInorm/mIdup>1.4.

Designation of pv.left and pv.right was iteratively determined by agreement of PennCNV and visual calls for each dataset. For example, in the NYCP dataset, to call the copy number genotypes as '3' pv.right cutoff was changed to pv.right<0.01.

In all datasets, an exception was made to criteria for genotype call of CN3 if nHalf=0 and if visual inspection revealed zero SNPs BAF=0.5±0.4, then the mInorm/mIdup cutoff was changed to ≥1.

Case/control QC for association: A subset of variants common to Genentech RA cases and Gene By Gene controls were analyzed to match the groups for European ancestry by PCA and analyze relatedness between individuals using Golden Helix SVS. Non-Europeans were excluded from the case/control groups for comparison of *SLC2A3* CNV frequencies. Individuals were removed from the study so that no two individuals had estimated relatedness closer than third degree.

CNV genotype frequencies between cases and controls odds-ratios (OR±95% confidence interval) were calculated according to Altman DG, 1991 (Practical statistics for medical research. London: Chapman and Hall.). CNV genotype frequencies between cases and controls P-value is calculated according to Sheskin, 2004 (Handbook of parametric and nonparametric statistical procedures. 3rd ed. Boca Raton: Chapman & Hall /CRC. p. 542). A standard normal deviate (z-value) is calculated as ln(OR)/SE{ln(OR)}, and the P-value is the area of the normal distribution that falls outside ±z.

MADGC family relatedness correction was performed as described in (Yang et al., 2011).

Graphs and statistical tests: of expression and glycolysis data was performed in GraphPad Prism V5.03 (GraphPad Software, Inc.). Specific tests are indicated in figure legends.

Table S2. Data from *SLC2A3* CNV specific calling algorithm, PennCNV, visual and Taqman genotyping.

Sample group	# subjects in group with these variables	Consensus *SLC2A3* copy number	qPCR CN	Visual call	SLC2A3 CNV specific calling algorithm: Copy number call	P-value t test left inside	sign (inside left)	P-value inside right	sign (inside right)	mlnorm/mldup	nHalf2	PennCNV: *SLC2A3* copy number	LRR SD	NumCNV	# SNPs
GAP	12	1	1	1	1	2.8E-11 (1.1E-16 - 2.9E-06)	-1	4.7E-11 (2.2E-16 - 3.3E-07)	-1	1.00	0	1	0.1656 (0.1279 - 0.344)	5 (2 - 35)	26
NYCP	2	1	1	1	1	6.6E-09, 2.2E-06	-1	2.9E-09, 1.2E-06	-1	1.00	0	1	0.1627, 0.1889	3, 4	26
GAP	5	1	1	1	1	1.0E-15 (1.0E-50 - 1.5E-10)	-1	1.4E-13 (1.0E-50 - 8.9E-10)	-1	1.00	0	1	0.1521 (0.1307 - 0.1981)	4 (2 - 7)	28
NYCP	2	1	1	1	1	4.1E-13, 6.6E-09	-1	5.2E-13, 9.4E-09	-1	1.00	0	1	0.182, 0.1329	24, 2	28
GAP	358	2	2	2	2	0.14 (0.0003 - 0.50)	-1	0.23 (0.0002 - 0.50)	-1	0.37 (0.09 - 1.92)	4 (0 - 9)	2	0.1604 (0.1109 - 0.471)	3 (0 - 82)	
NYCP	18	2	2	2	2	0.35 (0.095 - 0.50)	-1	0.39 (0.045 - 0.45)	1	0.26 (0.13 - 1.49)	3 (1 - 8)	2	0.1523 (0.1145 - 0.6738)	1 (0 - 41)	
GAP	482	2	2		2	0.22 (0.0002 - 0.50)	1	0.19 (3.8E-05 - 0.49)	1	0.37 (0.11 - 1.64)	4 (0 - 9)	2	0.1733 (0.1036 - 0.7355)	3 (0 - 59)	
GAP	232	2	2		2	0.30 (0.01 - 0.50)	-1	0.36 (0.07 - 0.50)	1	0.32 (0.14 - 1.58)	4 (0 - 10)	2	0.1604 (0.1087 - 0.3158)	3 (0 - 214)	
NYCP	192	2	2		2	0.13 (0.001 - 0.49)	1	0.20 (0.002 - 0.49)	1	0.30 (0.07 - 1.00)	4 (0 - 9)	2	0.1619 (0.1043 - 0.4347)	1 (0 - 61)	
NYCP	133	2	2		2	0.24 (0.002 - 0.50)	-1	0.14 (0.0008 - 0.50)	-1	0.29 (0.09 - 1.53)	4 (0 - 8)	2	0.1466 (0.1021 - 0.6909)	1 (0 - 47)	
NYCP	101	2	2		2	0.13 (0.001 - 0.49)	1	0.20 (0.002 - 0.49)	-1	0.30 (0.07 - 1.00)	4 (0 - 9)	2	0.1662 (0.1047 - 0.8879)	1 (0 - 61)	
GAP	2	3	3	3	3	4.7E-05 - 0.0014	1	7.1E-06 - 0.0003	1	1.62, 3.86	0	3	0.1406, 0.1548	4 - 6	25
GAP	3	3	3	3	3	2.8E-06 (1.8E-07 - 0.0006)	1	3.0E-05 (1.4E-07 - 8.2E-05)	1	3.19 (2.53 - 5.19)	0 (0 - 1)	3	0.1438 (0.1098 - 0.1461)	6 (4 - 21)	10
GAP	12	3	3	3	3	0.0003 (1.5E-07 - 0.0018)	1	3.4E-05 (1.4E-07 - 0.0004)	1	3.10 (2.34 - 3.57)	0 (0 - 1)	3	0.1565 (0.1319 - 0.2344)	4 (2 - 8)	24
GAP	5	3	3	3	3	2.8E-06 (1.8E-07 - 0.0005)	1	3.0E-05 (1.4E-07 - 8.2E-05)	1	3.19 (2.53 - 5.19)	0 (0 - 1)	3	0.1537 (0.1395 - 0.2053)	6 (4 - 21)	26
GAP	5	3	3	3	3	0.0001 (4.5E-07 - 0.0008)	1	6.2E-05 (2.6E-06 - 0.0002)	1	3.44 (2.54 - 5.24)	0 (0 - 1)	3	0.1533 (0.1273 - 0.1625)	5 (4 - 6)	27
NYCP	4	3	3	3	3	2.9E-10 (8.9E-13 - 1.04E-07)	1	2.1E-08 (4.9E-10 - 0.0002)	1	5.67 (2.85 - 6.50)	0	3	0.1850 (0.1248 - 0.2262)	14 (2 - 27)	28
GAP	3	3	3	3	3	2.8E-06 (1.8E-07 - 0.0006)	1	3.4E-05 (1.4E-07 - 8.2E-05)	1	3.19 (2.53 - 5.19)	0 (0 - 1)	3	0.2623 (0.2433 - 0.2647)	6 (4 - 21)	28
GAP	2	3	3	3	3	0.0005, 0.0009	1	0.0016, 0.00015	1	3.80, 4.58	0	3	0.1733, 0.158	3, 7	22
GAP	2	3	3	3	3	1.3E-08, 0.00002	1	6.3E-08, 1.5E-05	1	2.26, 4.70	0	3	0.1179, 0.1521	2, 6	23
GAP	4	3	3	3	3	0.0002 (0.0001 - 0.001)	1	7.1E-05 (9.5E-05 - 4.9E-05)	1	2.98 (1.41 - 3.60)	0 (0 - 1)	3	0.158 (0.1349 - 0.1714)	4 (2 - 5)	8
GAP	2	3	3	3	3	9.8E-08 - 0.004	1	1.0E-06, 0.0004	1	2.66 - 4.51	0	3	0.2076 - 0.1487	4 - 6	22
GAP	4	3	3	3	3	0.0007 (1.5E-08 - 0.0016)	1	1.3E-05 (5.6E-07 - 8.5E-05)	1	2.81 (2.13 - 2.98)	0	3	0.1727 (0.1417 - 0.2416)	4 (3 - 7)	26
GAP	2	3	3	3	3	2.4E-05, 0.0003	1	4.7E-05, 8.1E-05	1	2.56, 2.48	1	3	0.1452, 0.1459	2, 7	19
GAP	2	3	3	3	3	2.9E-05, 3.8E-05	1	6.3E-07, 6.8E-06	1	2.256, 3.13	1	3	0.1396, 0.1294	6, 3	23
GAP	3	3	3	3	3	0.00016 (2.3E-05 - 0.025)	1	0.0003 (5.4E-06 - 0.006)	1	4.16 (1.81 - 4.25)	0	3	0.1715 (0.1398 - 0.1793)	4 (4 - 8)	17
GAP	2	3	3	3	3	0.0002, 0.0012	1	4.2E-05, 0.0001	1	3.90, 2.52	0	3	0.1427, 0.1607	4, 4	20
GAP	2	3	3	3	3	5.9E-06, 0.0003	1	2.1E-06, 0.0002	1	9.04, 3.33	0	3	0.1824, 0.173	9, 6	21
NYCP	2	3	3		3	6.8E-11, 5.9E-11	1	1.37E-09, 1.22E-10	1	4.51, 2.84	0	3	0.1987, 0.1486	9, 2	26
GAP	1	1	1	1	1	3.4E-11	-1	3.6E-10	-1	1.00	0	1	0.1585	6	23
NYCP	1	1	1	1	1	7.8E-05	-1	5.9E-07	-1	1.00	0	1	0.249	5	24
GAP	1	1	1	1	1	8.6E-10	-1	4.1E-09	-1	1.00	0	1	0.1448	1	27
GAP	1	1	1	1	1	6.7E-12	-1	1.0E-10	-1	1.00	0	1	0.2133	9	24
GAP	1	3	3	3	3	7.0E-04	1	3.6E-05	1	3.34	0	3	0.1254	4	13
GAP	1	3	3	3	3	1.4E-03	1	1.0E-05	1	2.00	0	3	0.1735	9	29
GAP	1	3	3	3	3	1.6E-04	1	8.4E-05	1	3.62	0	3	0.1417	5	9
GAP	1	3	3	3	3	2.3E-05	1	3.9E-06	1	2.59	1	3	0.1185	6	12
GAP	1	3	3	3	3	1.3E-07	1	1.7E-05	1	2.83	0	3	0.1768	3	22
GAP	1	3	3	3	3	2.5E-05	1	1.5E-04	1	3.12	0	3	0.1369	7	9
GAP	1	3	3	3	3	7.0E-05	1	7.1E-05	1	2.39	1	3	0.2942	3	24
GAP	1	3	3	3	3	1.5E-04	1	1.3E-05	1	2.42	1	3	0.1329	4	22
GAP	1	3	3	3	3	1.3E-02	1	4.4E-04	1	2.96	0	3	0.1413	2	16
NYCP	1	3	3	3	3	5.7E-05	1	1.3E-05	1	2.32	0	3	0.163	11	17
GAP	1	3	3	3	3	8.7E-05	1	8.9E-05	1	4.48	0	3	0.2265	3	20
GAP	1	3	3	3	3	2.1E-03	1	4.1E-04	1	4.20	0	3	0.1695	4	12
GAP	1	3	3	3	3	7.8E-05	1	7.0E-06	1	3.86	0	3	0.1502	5	10
GAP	1	3	3	3	3	1.7E-03	1	1.9E-04	1	3.59	0	3	0.1643	5	5
NYCP	1	3	3		3	9.6E-10	1	5.5E-09	1	3.15	0	3	0.144	14	22
NYCP	1	3	3		3	3.4E-11	1	6.8E-11	1	5.76	0	3	0.127	1	27
NYCP	1	3	3		3	1.2E-10	1	2.9E-09	1	1.90	0	3	0.239	8	17
NYCP	1	3	3		3	5.6E-08	1	5.8E-08	1	5.07	0	3	0.193	1	24
NYCP	1	3	3		3	1.1E-07	1	1.4E-07	1	3.82	0	3	0.131	1	25
NYCP	1	3	3		3	4.0E-04	1	1.9E-05	1	4.25	0	3	0.146	1	26
NYCP	1	3	3		3	4.0E-04	1	1.4E-04	1	3.17	0	3	0.168	12	21

PennCNV intervals (hg19):

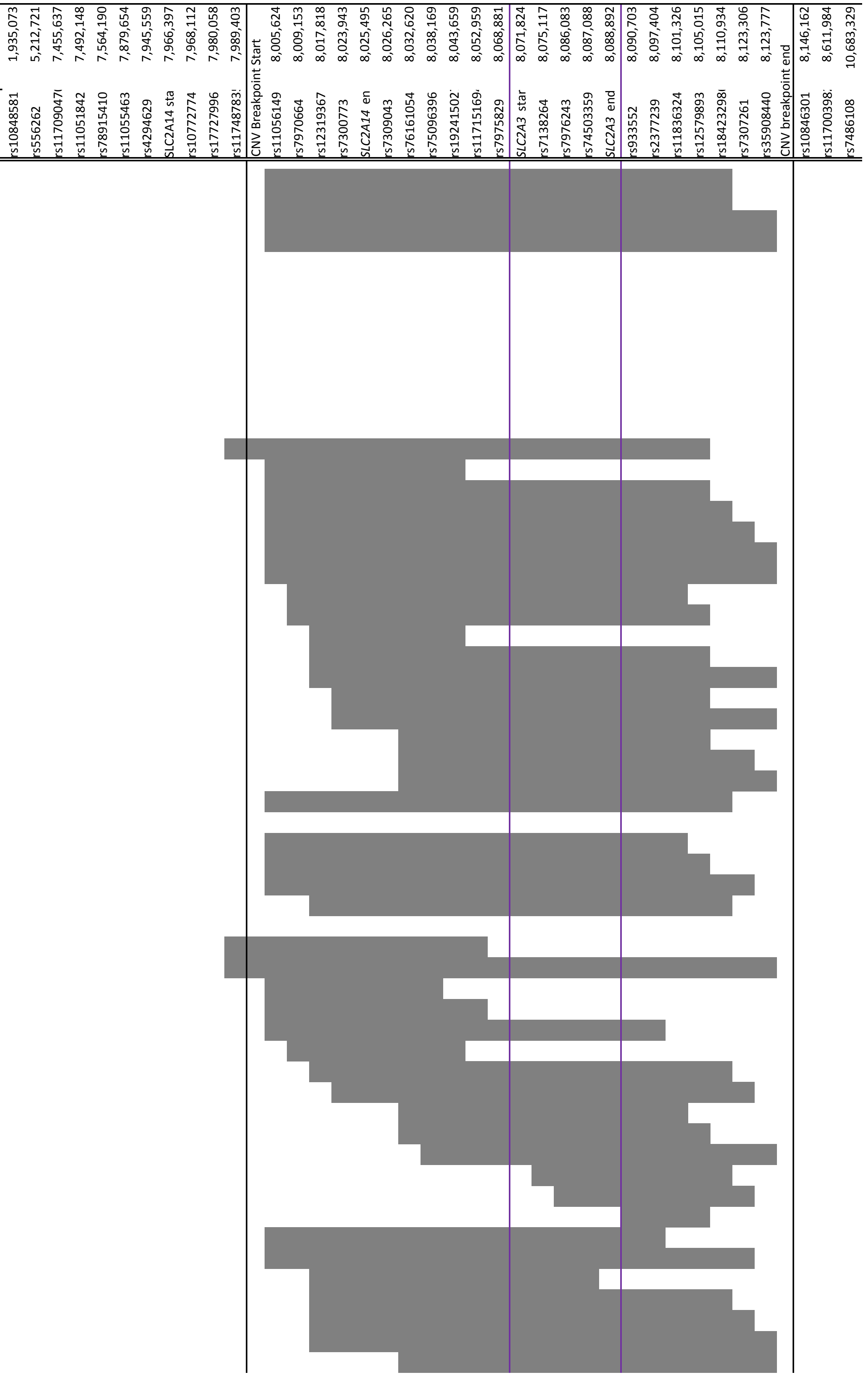

Sample group	# subjects in group with these variables	Consensus *SLC2A3* copy number	qPCR CN	Visual call	SLC2A3 CNV specific calling algorithm: Copy number call	P-value t test left inside	sign (inside left)	P-value inside right	sign (inside right)	mlnorm/mldup	nHalf2	PennCNV: *SLC2A3* copy number	LRR SD	NumCNV	# SNPs
NYCP	1	3	3		3	1.7E-07	1	1.7E-07	1	3.47	0	3	0.216	2	15
NYCP	1	3	3		3	2.2E-06	1	2.2E-07	1	2.93	0	3	0.179	1	14
NYCP	1	4	4	3	3	2.4E-13	1	4.5E-13	1	2.35	0	4	0.181	13	26
GAP	1	1	1	1	1	3.7E-06	-1	4.6E-06	-1	1.15	0	2*	0.2047	31	
GAP	1	3	3	3	3	1.5E-06	1	2.7E-06	1	2.43	1	2*	0.2463	4	
GAP	1	3	3	3	3	9.1E-05	1	2.2E-03	1	3.10	0	2*	0.1816	1	
GAP	1	3	3	3	3	2.0E-04	1	5.6E-04	1	1.96	1	2*	0.2709	4	
GAP	1	3	3	3	3	5.4E-04	1	4.1E-02	1	2.02	0	2*	0.3168	25	
NYCP	1	3	3	3	3	1.5E-02	1	9.7E-03	1	2.45	0	2*	0.175	0	
NYCP	1	3	3	3	3	1.6E-05	1	1.2E-05	1	1.53	0	2*	0.194	14	
NYCP	1	3	3	3	3	3.9E-09	1	4.6E-08	1	1.00	0	2*	0.168	27	
GAP	1	2	2	2	2	1.9E-02	-1	1.5E-02	1	0.33	7	3*	0.1216	2	27
GAP	1	2	2	2	2	3.9E-01	1	3.9E-01	-1	1.51	0	3*	0.2403	145	16
GAP	1	2	2	2	2	1.6E-01	1	2.2E-01	1	1.12	2	3*	0.1562	1	5
NYCP	2	2	2	fail	2	0.29, 0.45	-1	0.27, 0.36	1	1.43, 1.49	4, 4	2	0.6738, 0.6307	20, 41	
NYCP	1	2	2	fail	2	3.0E-01	1	5.0E-01	-1	1.56	0	2	0.817	120	34
NYCP	1	2	2	fail	2	3.9E-01	-1	4.5E-01	-1	1.61	0	1*	0.699	168	979
NYCP	1	2	2	fail	2	8.9E-02	1	1.3E-01	1	1.01	0	1*	0.821	117	162
NYCP	1	2	2	fail	2	3.7E-01	-1	3.4E-01	-1	1.04	1	1*	0.738	26	32
GAP	1	2	2	fail	2	3.8E-01	1.00	7.0E-02	-1.00	1.52	1	3*	0.6042	35	2822
NYCP	1	2	2	fail	2	3.0E-01	1	1.0E-01	-1	1.13	4	3*	0.731	12	96
GAP	1	2	2	fail	2	1.2E-01	-1.00	2.2E-01	1.00	2.37	0	3*	0.1703	191	19

PennCNV intervals (hg19):

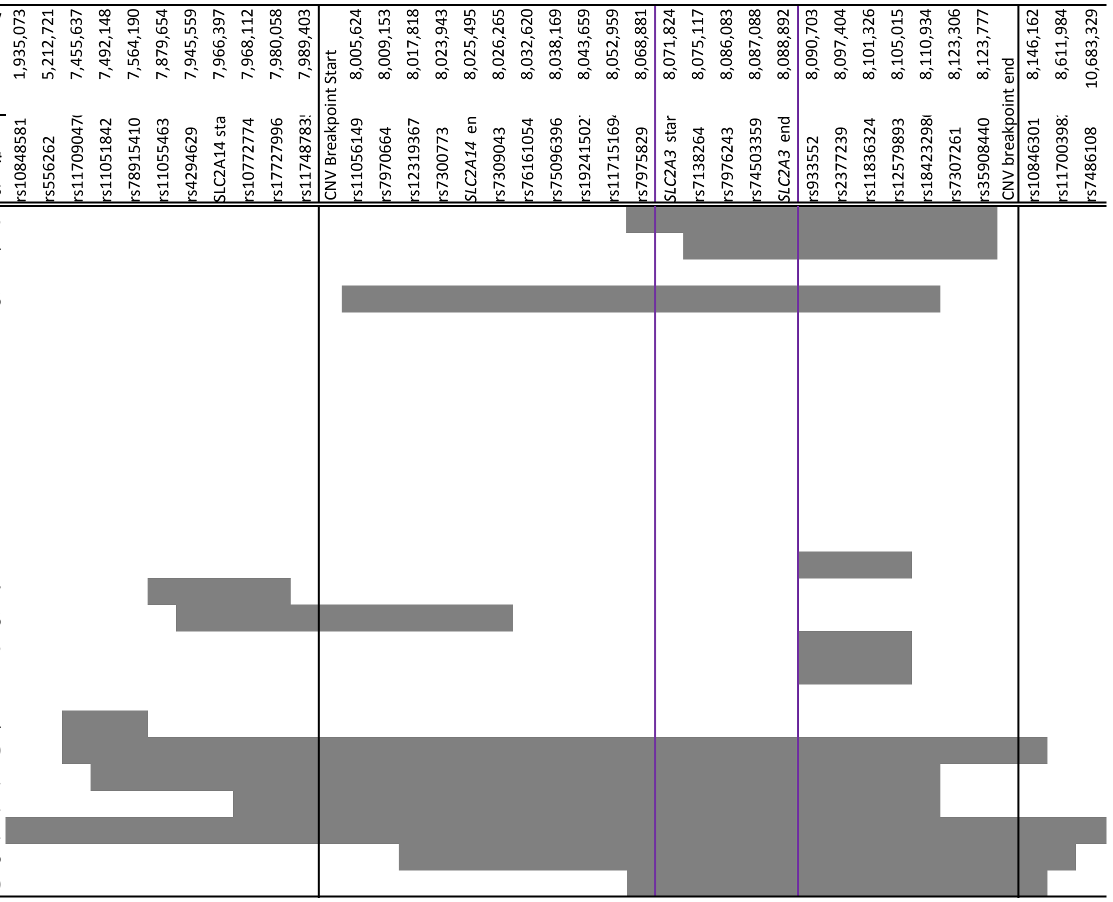

Table S3. *SLC2A3* CNV genotype data in cases and control groups.

Group	Cohort	Group	Sub group	# SNPs				Number passed QC	Copy number count				Frequency of copy number		
				Chr12	left*	inside*	right*		1	2	3	4	1	2	≥3
1	Genentech	RA cases	**Sum**	-	-	-	-	**5,135**	**40**	**4873**	**222**	**0**	**0.78**	**94.90**	**4.32**
			a	53,878	153	52	163	1,965	15	1,864	86	0	0.76	94.86	4.38
			b	112,722	309	107	201	306	2	292	12	0	0.65	95.42	3.92
			c	113,214	311	106	208	1,942	12	1,847	83	0	0.62	95.11	4.27
			d	27,143	75	24	39	712	10	665	37	0	1.40	93.40	5.20
			e	57,391	153	54	131	210	1	205	4	0	0.48	97.62	1.90
	GxG	Controls	**Sum**	-	-	-	-	**14,145**	**121**	**13390**	**634**	**0**	**0.86**	**94.66**	**4.48**
			a	10,705	100	33	61	11,075	104	10,479	492	0	0.94	94.62	4.44
			b	10,864	104	36	61	3,070	17	2,911	142	0	0.55	94.82	4.63
2	Spanish	RA cases	**Sum**	-	-	-	-	**3438**	**23**	**3257**	**155**	**3**	**0.67**	**94.74**	**4.60**
			a	14,807	59	20	37	2,702	18	2,567	115	2	0.67	95.00	4.33
			b	14,807	59	20	37	736	5	690	40	1	0.68	93.75	5.57
		Control	**Sum**	-	-	-	-	**1359**	**9**	**1284**	**63**	**3**	**0.66**	**94.48**	**4.86**
			a	14,807	59	20	37	1,010	6	959	43	2	0.59	94.95	4.46
			b	57,391	153	54	131	349	3	325	20	1	0.86	93.41	5.73
3	RACI	RA cases	-	-	-	-	-	**1,798**	**10**	**1,706**	**78**	**4**	**0.56**	**94.88**	**4.56**
		Controls	-	-	-	-	-	**1,870**	**13**	**1,787**	**69**	**1**	**0.70**	**95.56**	**3.74**
4	Korean	RA cases	**Sum**	-	-	-	-	**1,170**	**11**	**1,096**	**61**	**2**	**0.94**	**93.68**	**5.38**
			a	29,330	87	33	55	793	10	745	39	0	1.26	93.95	4.92
			b	29,330	87	33	55	12	0	11	1	0	0.00	91.67	8.33
			c	-	-	-	-	365	1	340	21	2	0.27	93.15	6.30
		Controls	**Sum**	-	-	-	-	**892**	**9**	**851**	**32**	**0**	**1.01**	**95.40**	**3.59**
			a	29,330	87	33	55	380	1	368	12	0	0.26	96.84	3.16
			b	29,330	87	33	55	112	0	110	4	0	0.00	98.21	3.57
			c	-	-	-	-	400	8	375	17	0	2.00	93.75	4.25
5	MADGC	RA cases	-	**32,563**	**75**	**26**	**50**	**259**	**3**	**250**	**6**	**0**	**1.16**	**96.53**	**2.32**
		MS Cases	-	**32,563**	**75**	**26**	**50**	**260**	**7**	**241**	**12**	**0**	**2.69**	**92.69**	**4.62**
6	WTCCC2	MS Cases	-	**31,680**	**93**	**32**	**132**	**10,508**	**69**	**9946**	**493**	**0**	**0.66**	**94.65**	**4.69**
		Controls	-	**31,680**	**93**	**32**	**132**	**663**	**3**	**632**	**28**	**0**	**0.45**	**95.32**	**4.22**

***Left of CNV interval (hg19 chr12: 7,645,630 - 7,995,630), Inside of CNV interval (hg19 chr12: 7,995,630 - 8,125,390), Right of CNV interval (hg19 chr12: 8,125,390 - 8,475,390)**

Table S4. Summary of the false positive and false negative PennCNV calls.

Cohort		Group		Number samples analyzed	Number passed QC	PennCNV accurate	Actual CN	1		2		2		3		Fail		Fail		Fail		Failed Max LRR SD	Failed Max Num CNV	Passed Max LRR SD	Passed Max Num CNV
							PennCNV	2		1		3		2		1		2		3					
								#	%	#	%	#	%	#	%	#	%	#	%	#	%				
1	Genentech	RA	**Sum**		**5,135**																				
			a	2,928	1,965	2827		15	0.51	0	0.00	4	0.14	73	2.50	0	0.00	7	87.50	1	12.50	0.2913	399	0.35	156
			b	384	306	364		4	1.04	2	0.52	0	0.00	14	3.65	0	0.00	0	0.00	0	0.00			0.5201	237
			c	2,557	1,942	2411		14	0.55	59	2.33	1	0.04	48	1.89	0	0.00	14	58.33	10	41.67	0.9252	688	0.5782	297
			d	959	712	921		5	0.52	0	0.00	0	0.00	33	3.44	0	0.00	1	100.00	0	0.00			0.3982	31
			e	255	210	255		0	0	0	0	0	0	0	0	0	0.00	0	0.00	0	0.00			0.3085	7
	GxG	Con	**Sum**		**14,145**																				
			a	11,075	11,075	11053		0	0.00	1	0.01	2	0.02	19	0.17	0	0.00	0	0.00	0	0.00			0.4941	16
			b	3,070	3,070	3066		0	0.00	0	0.00	0	0.00	4	0.13	0	0.00	0	0.00	0	0.00			0.5066	11
2	Spanish	RA	**Sum**		**3438**																				
			a	2,891	2,702	2707		1	0.04	0	0.00	4	0.15	9	0.33	0	0.00	166	97.65	4	2.35	1.1077	221	0.4038	35
			b	748	736	732		0	0.00	1	0.14	1	0.14	2	0.27	0	0.00	12	100.00	0	0.00	0.7396	16	0.3694	26
		Con	**Sum**		**1359**																				
			a	1,044	1,010	1008		1	0.10	0	0.00	0	0.00	1	0.10	0	0.00	32	94.12	2	5.88	0.5623	170	0.3675	61
			b	397	349	349		0	0.00	0	0.00	0	0.00	0	0.00	2	4.00	13	26.00	35	70.00	0.957	518	0.4573	88
4	Korean	RA	**Sum**		**1,170**																				
			a	806	793	793		0	0.00	1	0.13	0	0.00	0	0.00	0	0.00	0	0.00	0	0.00			0.5896	10
			b	15	12	11		0	0.00	0	0.00	0	0.00	1	8.33		0.00	1	33.33	2	66.67	0.6264	261	0.359	4
		Con	**Sum**		**892**																				
			a	391	380	371		0	0.00	10	2.62	0	0.00	0	0.00	0	0.00	0	0.00	0	0.00			0.6223	85
			b	122	112	97		0	0.00	15	13.16	0	0.00	2	1.75	2	20.00	7	70.00	1	10.00	0.9365	123	0.6846	106
6	WTCCC2	MS	-	**10,667**	**10,508**	**10470**		**1**	**0.01**	**1**	**0.01**	**3**	**0.03**	**33**	**0.31**	**1**	0.65	**150**	96.77	**4**	2.58	**0.7109**	**371**	**0.545**	**66**
		Con	-	**709**	**663**	**659**		**0**	**0.00**	**0**	**0.00**	**0**	**0.00**	**4**	**0.60**	**0**	0.00	**43**	97.73	**1**	2.27	**0.5942**	**323**	**0.4488**	**49**

Table S5. Population frequencies of *SLC2A3* CNV.

Ancestry from PCA	Self-reported ancestry #	Self reported ethnicity	Count (%) subjects by *SLC2A3* gene copy number 1	2	≥ 3	Total
**EUR**			**1.27%** **26**	**93.88%** **1,919**	**4.84%** **99**	**2,044**
EUR			21	1,471	79	1,571
EUR		Ashkenazi Jewish	4	347	11	362
NA	EUR			58	8	66
EUR		Latino/Hispanic	1	33	1	35
NA	EUR	Ashkenazi Jewish		10		10
**AFR**			**0.63%** **2**	**95.56%** **301**	**3.81%** **12**	**315**
AFR			2	259	11	272
AFR		Latino/Hispanic		31	1	32
NA	AFR			11		11
**EAS**			**0.89%** **1**	**96.43%** **108**	**2.68%** **3**	**112**
EAS			1	103	3	107
NA	EAS			4		4
EAS		Latino/Hispanic		1		1
**SAS**			**0.00%** **-**	**95.93%** **118**	**4.07%** **5**	**123**
SAS				110	5	115
NA	SAS			5		5
SAS		Latino/Hispanic		3		3
**AMR**			**0.68%** **1**	**98.64%** **145**	**0.68%** **1**	**147**
AMR		Latino/Hispanic		124	1	125
AMR			1	17		18
AMR		Ashkenazi Jewish		2		2
NA	AMR	Latino/Hispanic		2		2

if no PCA or if different from PCA

Table S6. Power to detect original protective association data in the sample groups sizes of this study. Values were calculated using Sampsize (http://sampsize.sourceforge.net/), using the parameters shown.

Power calculation parameters	Group	Cohort	Group	Total number subjects	Number of controls per case	Power to replicate protective association in Swedish RA cases and controls (Veal *et al* Table 1)		Power to replicate protective association in UK & US RA cases and controls (Veal *et al* Table 2)	
**Odds Ratio**						**0.442**	**0.442**	**0.559**	**0.559**
**Frequency of deletion in controls**						**2.60%**	**2.60%**	**0.905%**	**0.905%**
**Tailed test**						**Two**	**One**	**Two**	**One**
**Alpha Risk**						**5**	**5**	**5**	**5**
	1	Genentech GxG	RA cases<br>Controls	5,135<br>14,145	2.75	99.9999	100	81.2	89.3
	2	Spanish	RA cases<br>Control	3438<br>1359	0.395	91.8	95.2	36.8	47.6
	3	RACI	RA cases<br>Controls	1,798<br>1,870	1.04	89.1	94.0	29.8	41.5
	4	Korean	RA cases<br>Control	1,170<br>892	0.76	67.5	77.4	20.0	29.6

Table S7. Test for sub-group heterogeneity.

Group	Cohort	Group	Sub group	Number passed QC	Copy number count 1	Copy number count 2	Copy number count 3	Copy number count 4	Frequency of copy number 1	Frequency of copy number 2	Frequency of copy number ≥3	Pearson's Chi-square 1 vs 2 vs 3 or more copies: X-Squared	Pearson's Chi-square 1 vs 2 vs 3 or more copies: degrees of freedom	Pearson's Chi-square 1 vs 2 vs 3 or more copies: p-value	Pearson's Chi-square (*Yate's continuity correction for 2x2) 1 vs 2 or more copies: X-Squared	Pearson's Chi-square (*Yate's continuity correction for 2x2) 1 vs 2 or more copies: degrees of freedom	Pearson's Chi-square (*Yate's continuity correction for 2x2) 1 vs 2 or more copies: p-value
1	Genentech	RA cases	a	1,965	15	1,864	86	0	0.76	94.86	4.38	9.1	8	0.33	4.6	4	0.33
			b	306	2	292	12	0	0.65	95.42	3.92						
			c	1,942	12	1,847	83	0	0.62	95.11	4.27						
			d	712	10	665	37	0	1.40	93.40	5.20						
			e	210	1	205	4	0	0.48	97.62	1.90						
	GxG	Controls	a	11,075	104	10,479	492	0	0.94	94.62	4.44	4.4	2	0.11	3.8	1	0.052 *
			b	3,070	17	2,911	142	0	0.55	94.82	4.63						
2	Spanish	RA cases	a	2,702	18	2,567	115	2	0.67	95.00	4.33	2.0	2	0.36	0.002	1	0.97 *
			b	736	5	690	40	1	0.68	93.75	5.57						
			a	1,010	6	959	43	2	0.59	94.95	4.46	1.7	2	0.43	0.021	1	0.89 *
			b	349	3	325	20	1	0.86	93.41	5.73						
4	Korean	RA cases	a	793	10	745	39	0	1.26	93.95	4.92	3.8	4	0.43	2.7	2	0.26
			b	12	0	11	1	0	0.00	91.67	8.33						
			c	365	1	340	21	2	0.27	93.15	6.30						
		Controls	a	380	1	368	12	0	0.26	96.84	3.16	8.0	4	0.09	7.2	2	0.027
			b	112	0	110	4	0	0.00	98.21	3.57						
			c	400	8	375	17	0	2.00	93.75	4.25						
**All EUR controls**			GxG a	11,075	104	10479	492	0	0.94	94.62	4.44	11.3	10	0.33	6.8	5	0.24
			GxG b	3,070	17	2911	142	0	0.55	94.82	4.63						
			Spanish a	1,010	6	959	43	2	0.59	94.95	4.46						
			Spanish b	349	3	325	20	1	0.86	93.41	5.73						
			RACI	1,870	13	1787	69	1	0.70	95.56	3.74						
			WTCCC2	663	3	632	28	0	0.45	95.32	4.22						

Figure S1: RA clinical covariates are not influenced by *SLC2A3* gene copy number. RA clinical measures of Disease Activity Score in 28 Joints (A), Rheumatoid Factor (B), Erythrocyte Sedimentation Rate (C) and C-reactive protein (D) are plotted according to genotype of *SLC2A3* gene copy number. Each symbol represents one RA patient, CN1 n=21, CN2 n=6,156, CN3 n=411. Error bars represent median ± interquartile range. One-way ANOVA Kruskal-Wallis test was performed and P values are shown on each respective graph.

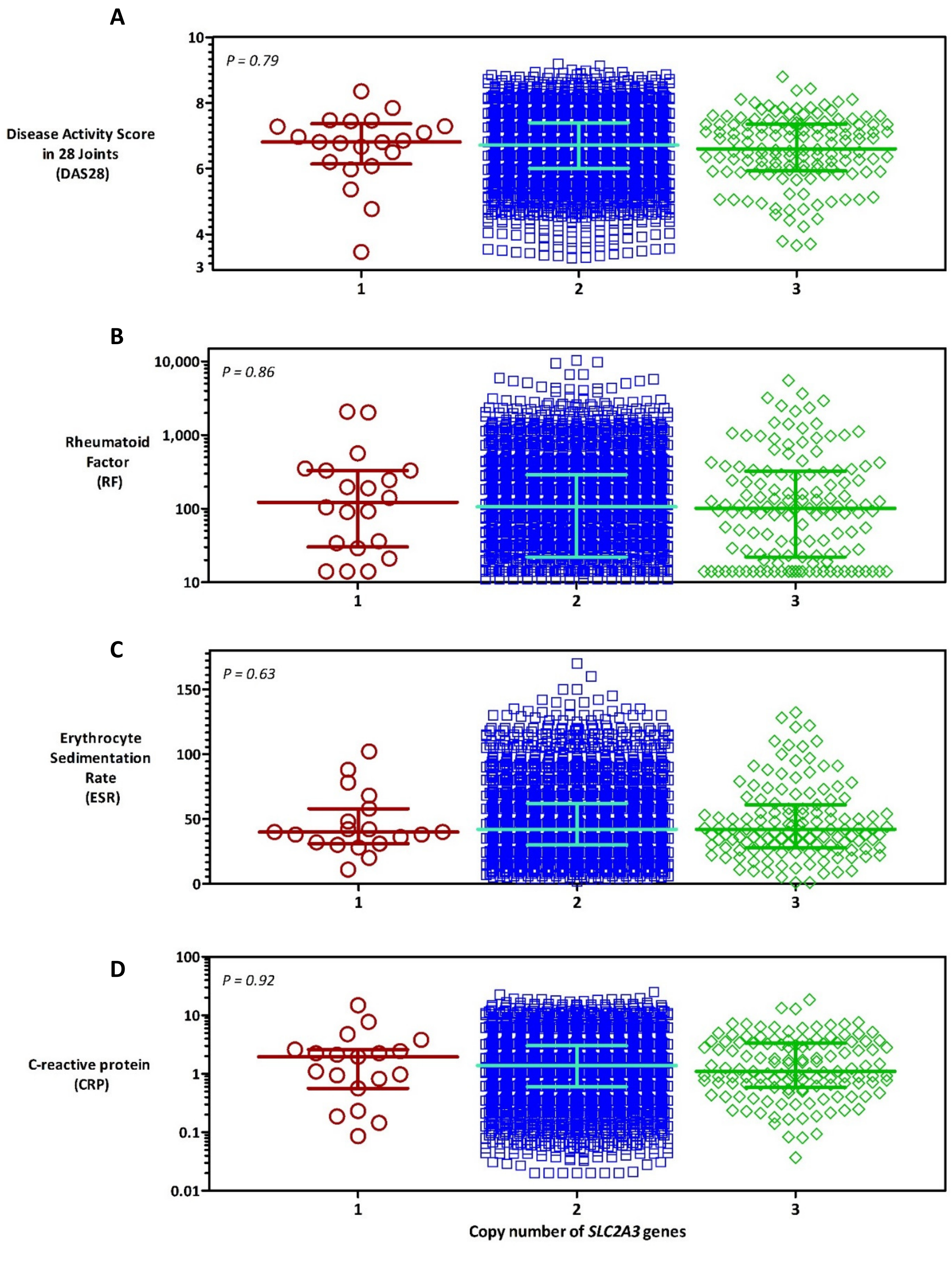

Figure S2: Validation of GLUT3 specific antibody and expression of GLUT3 protein in leukocyte subsets. A) HEK293T cells were transfected with constructs for expression of *SLC2A1*, *SLC2A3* and *SLC2A14* turboGFP tagged. GFP positive cells were sorted and lysates were run on Western blot and probed with the indicated primary antibodies. A rabbit polyclonal antibody to GLUT3 (ab32551) recognizes both GLUT3 and GLUT14, whereas a rabbit monoclonal antibody to the N-terminal of GLUT3 (EPR10508) is specific to GLUT3, and does not cross-react with GLUT14 or GLUT1. Note that lysates were boiled prior to loading on 4-20% gel, leading to large molecular weight smears for the recombinant GLUT-GFP proteins. The rabbit monoclonal also recognizes endogenous GLUT3 at the expected molecular weight of ~54kDa. **B)** Leukocyte subsets were isolated from PBMC or fresh blood (neutrophils) and lysed immediately, activated as indicated, or differentiated/expanded for indicated time points. Lysates were run in a 10% gel and membranes were probed with anti-GLUT3 (EPR10508(N)), followed by anti-GAPDH. GLUT3 bands were expected at ~54kDa and were observed at 58kDa (band 1) and 52kDa (band 2). GAPDH was expected at 38kDa and observed at 37kDa.

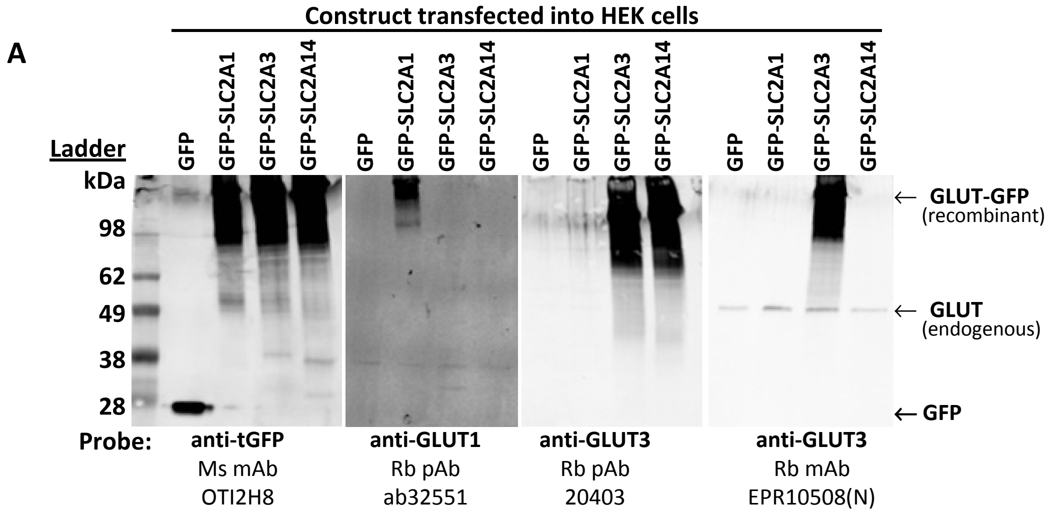

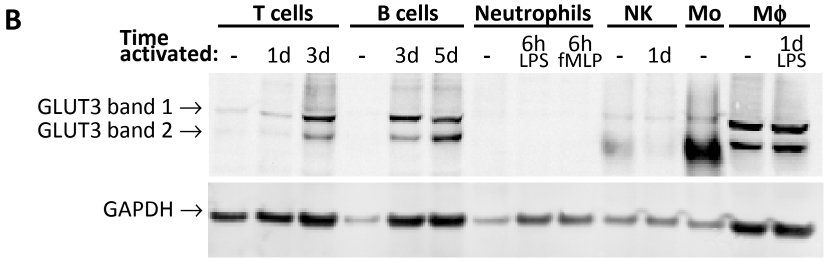

Figure S3: Gene expression of *SLC2A3* is influenced by gene deletion. Expression of *SLC2A3*, *SLC2A1* and neighboring genes was measured by qPCR in T-blasts (T) and macrophages (Mϕ) from genotyped subjects. A) *SLC2A3* CN1 versus CN2, B) *SLC2A3* CN2 versus CN3. Statistical significance was determined by Mann-Whitney U one-tailed t-test, * denotes *P<0.05*.

A

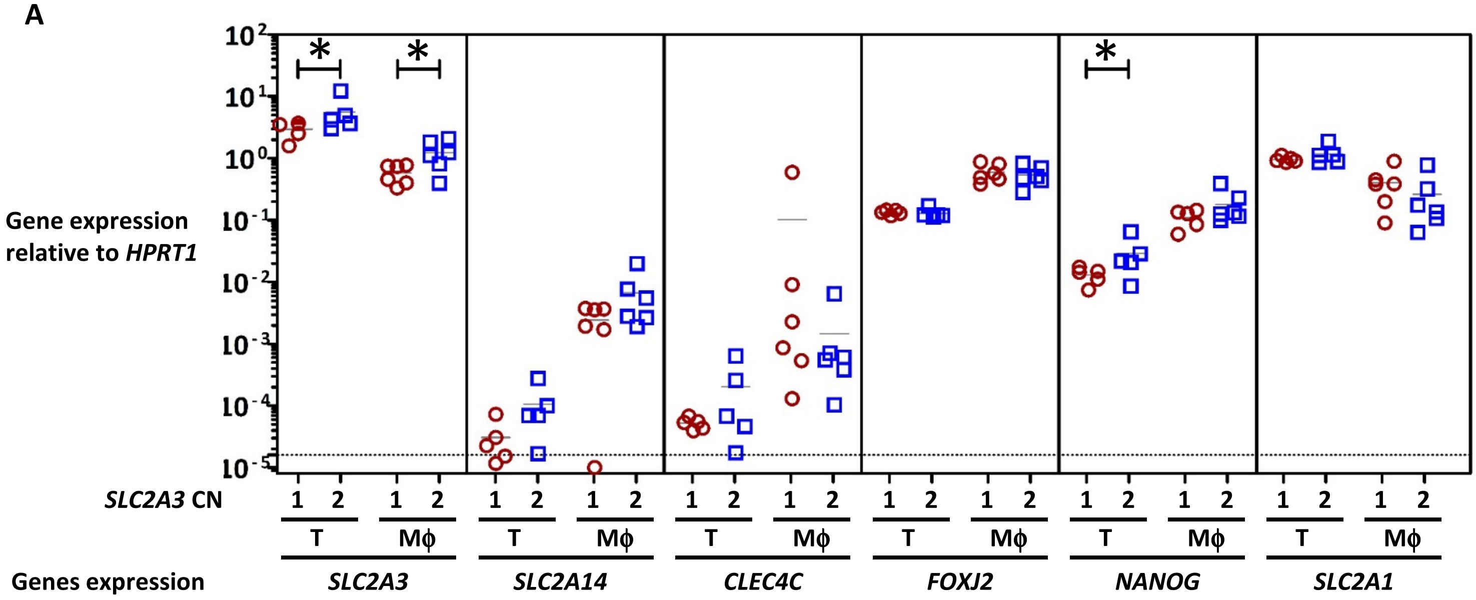

B

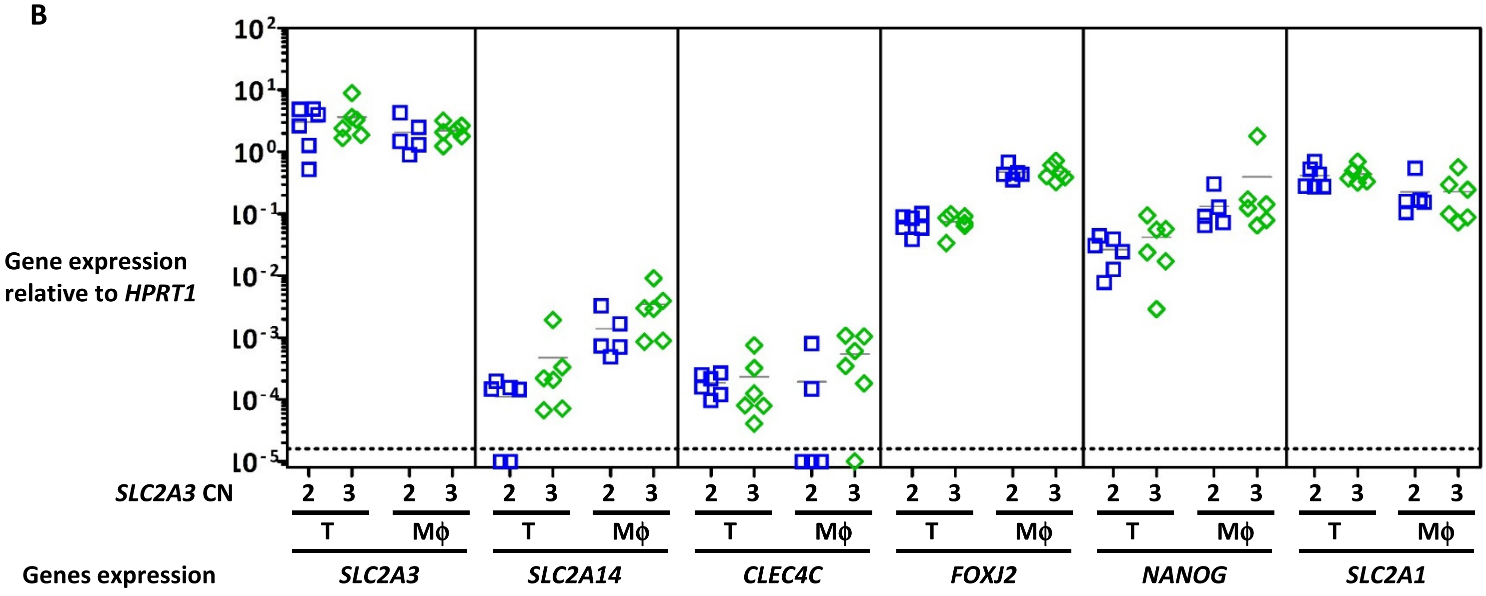

Figure S4: CNV genotyping by paralogue ratio test qPCR is prone to errors. A) The AH1SEM4 qPCR assay design is shown. This assay takes advantage of sequence similarity between *SLC2A3* and it's homologue *SLC2A14*. The probes detect a one base pair difference in the sequences of the two genes. Fold change in threshold cycle value by qPCR reflects the change in allele numbers present between the SLC2A3 allele G (inside the CNV interval), compared with SLC2A14 allele A (outside the CNV boundaries). **B)** The AH1SEM4 genotyping assay was performed in 861 GAP subjects, who were also genotyped by commercial TaqMan CNV assay and GSA chip methods. Expected genotype copy numbers were not corroborated by the AH1SEM4 assay in 42/861 subjects, illustrating that PRT assays produce spurious results in ~5% of samples.

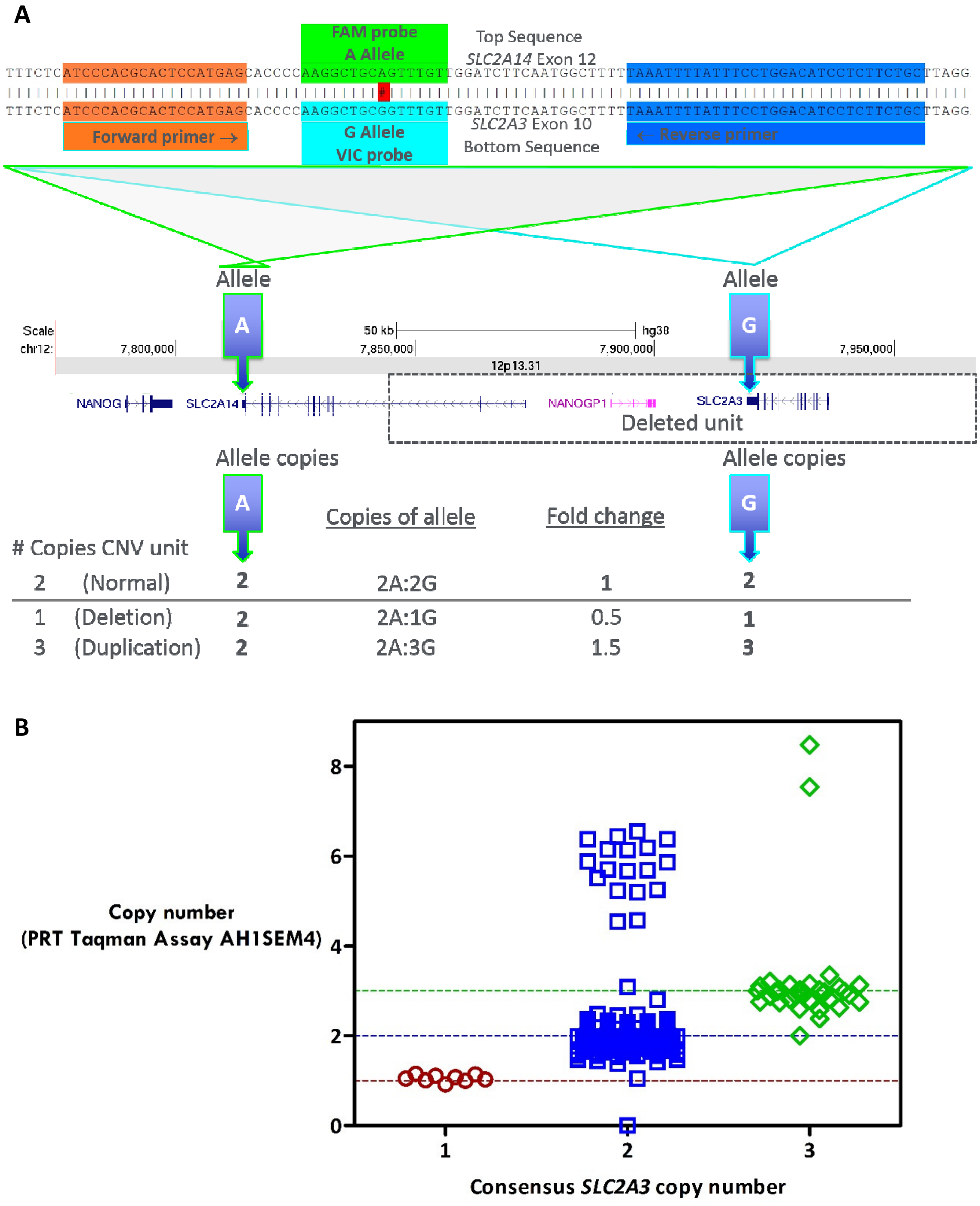

Figure S5: GLUT3 protein expression in T-blasts by *SLC2A3* gene copy number. T-blast lysates of genotyped subjects were analyzed by Western blot. GLUT3 band volumes were measured and are plotted relative to the band volume of the GAPDH loading control. CN genotypes were compared for each band by Mann Whitney two-tailed t-test and P values are given. A) lysates of day 7 T-blasts of CN2 and CN3 subjects loaded on a 4-20% gradient gel, B) lysates of day 7 T-blasts of CN1 and CN2 subjects loaded on a 4-20% gradient gel, C) lysates of day 10 T-blasts of CN1 and CN2 subjects loaded on a 10% gel, D) **boiled** lysates of day 10 T-blasts of CN1 and CN2 subjects loaded on a 4-20% gradient gel.

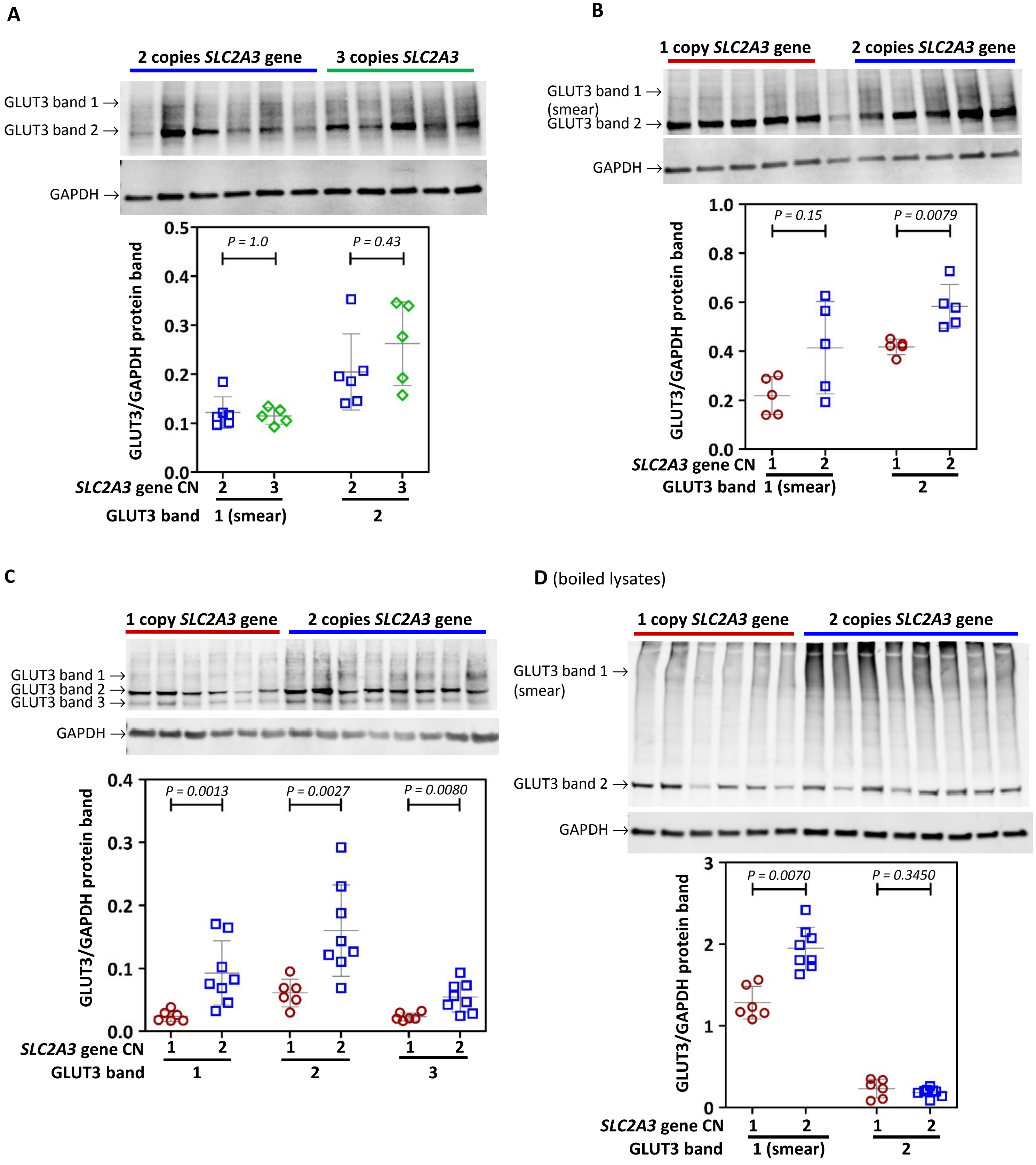

Supplement: Supplementary file 1 — Supplementary material [file mmc1.pdf]
